# Neighborhood Deprivation and Days Spent at Home After Fall-Related Hip Fracture

**DOI:** 10.1001/jamanetworkopen.2025.49118

**Published:** 2025-12-23

**Authors:** Alyssa M. Baginski, Robynne G. Braun, Chixiang Chen, Na Sun, Jay Magaziner, C. Daniel Mullins, Jason R. Falvey

**Affiliations:** 1 University of Maryland School of Medicine, Baltimore; 2 Division of Rehabilitation Medicine, Department of Neurology, University of Maryland School of Medicine, Baltimore; 3 Department of Epidemiology and Public Health, University of Maryland Medical School, Baltimore; 4 Department of Physical Therapy and Rehabilitation Science, University of Maryland Medical School, Baltimore; 5 Department of Practice, Sciences, and Health Outcomes Research, University of Maryland School of Pharmacy, Baltimore

## Abstract

**Importance:**

Hip fracture is a highly disabling event for older adults and increases the risk of needing prolonged care in health care facilities. Older adults in economically deprived neighborhoods may have fewer resources to support aging in place vs in a facility, but how neighborhood deprivation is associated with home time after hip fracture is unknown.

**Objective:**

To evaluate the association between neighborhood deprivation and days spent at home after hip fracture.

**Design, Setting, and Participants:**

This cohort study analyzed a random sample of Medicare claims and assessment data for beneficiaries who experienced a fall-related hip fracture from July 1, 2010, to December 31, 2019; underwent surgery (arthroplasty or internal fixation); and were discharged alive to a nonhospice home or postacute care setting. Neighborhood deprivation was assessed using the Area Deprivation Index. Data were analyzed from January 2023 to September 2025.

**Exposure:**

Area Deprivation Index (ADI; range, 1-100), where the 10th percentile or lower was categorized as the least deprived, the 11th to 89th percentile as moderately deprived, and the 90th to 100th percentile as the most deprived.

**Main Outcomes and Measures:**

Days at home in the 12 months after hip fracture.

**Results:**

The final analytical sample consisted of 52 012 older adults (mean [SD] age, 82.2 [8.1] years; 38 360 women [73.8%]) with a fall-related hip fracture. Those living in more deprived neighborhoods were more likely than those in the least deprived neighborhoods to identify as a member of a racial or ethnic minority group (Black or Hispanic, 477 of 4306 [11.1%] vs 120 of 5112 [2.3%]) and to be dually eligible for Medicaid (113 of 4306 [26.3%] vs 590 of 5112 [11.5%]). Relative to adults living in the least deprived areas, those in the most deprived areas spent 8.5% fewer days at home (incidence rate ratio [IRR], 0.92; 95% CI, 0.89-0.94), and those in the middle ADI group spent 5% fewer days at home (IRR, 0.95; 95% CI, 0.94-0.97). For patients in the most deprived neighborhoods, this resulted in an absolute difference of 22.8 days at home over the following year.

**Conclusions and Relevance:**

In this cohort study of older adults with a fall-related hip fracture, residence in an economically deprived area was associated with fewer days at home in the 12 months after the fracture. These findings suggest neighborhood context is an important determinant of postfracture recovery and could guide development of more community-tailored interventions.

## Key Points

**Question:**

Is neighborhood socioeconomic status associated with days spent at home after fall-related hip fracture among older Medicare beneficiaries?

**Findings:**

In this cohort study of 52 012 older adults with a fall-related hip fracture, higher neighborhood socioeconomic deprivation, as measured by the Area Deprivation Index, was negatively associated with days spent at home after hip fracture over 12 months.

**Meaning:**

These results suggest the socioeconomic context of the neighborhood environment is associated with home time for Medicare beneficiaries after a fall-related hip fracture.

## Teaser

This cohort study uses Medicare claims data to evaluate the association between neighborhood socioeconomic deprivation and days spent at home after fall-related hip fracture.

## Introduction

Every year, more than 300 000 people 65 years of age or older experience a hip fracture, with 88% of those cases resulting from falls.^1^ Hip fractures and other fall-related injuries contribute to an increasing number of deaths among older adults every year and are associated with nearly $20 billion in health care spending annually.^2,3^ Fewer than 35% of patients return to prior levels of activities of daily living independence or mobility status,^4^ and 10% to 20% require long-term care in the year after fracture, ^5^ which is not concordant with the major goal of aging in place for older adults recovering from serious illnesses and injuries.^6,7^ Although several medical and social factors have been associated with aging in place after hip fracture,^8,9,10^ there is a paucity of research evaluating the association of neighborhood socioeconomic context. Prior work suggests that living in a socioeconomically deprived neighborhood is negatively associated with disability-free life expectancy among community-dwelling older adults and those recovering from other fall-related injuries, such as traumatic brain injury.^11,12^ Long-term structural inequalities, including lower-quality built environment, food availability, and higher crime rates, may also be negatively associated with health outcomes for older adults with new disability.^13^

In the present study, we evaluated whether days spent at home after hip fracture, an emerging claims-based patient-centered outcome that reflects patient priorities for aging in place, differed by neighborhood socioeconomic status. We hypothesized that older adults with hip fracture living in areas of greater deprivation would spend fewer days at home. The results from this study could inform more tailored interventions to promote aging in place after hip fracture.

## Methods

### Data Source

The cohort study used standard Centers for Medicare & Medicaid Services (CMS) research identifiable files, including the annual 5% random sample of claims and assessment data for Medicare fee-for service (FFS) Part A and B beneficiaries from 2010 to 2016 and annual 20% random samples from 2017 to 2019. We excluded those who switched between FFS Medicare and Medicare Advantage plans during the study period, given that the CMS did not make Medicare Advantage encounter data before 2015 available to researchers. Fall-related hip fracture index admissions occurring between July 1, 2010, and December 31, 2019, were identified through the Medicare Provider and Analysis Review (MEDPAR) file. Additional data were gathered from the Medicare Master Beneficiary Summary File, Minimum Data Set (MDS) 3.0, Chronic Conditions, outpatient claims, and carrier claims to capture demographic information, comorbidities, geographic residence (eg, rural and urban as defined using Rural Urban Continuum Codes linked at the county level of the patient’s last documented residence prior to fracture), and health care use. This analysis of Medicare claims was designated as exempt, nonhuman participant research by the University of Maryland Baltimore institutional review board. Results were reported in accordance with the Strengthening the Reporting of Observational Studies in Epidemiology (STROBE) reporting guideline.

### Study Sample

Our cohort included adults older than 65 years who underwent surgery (arthroplasty or internal fixation^14^) for a fall-related hip fracture and were then discharged to inpatient rehabilitation settings, skilled nursing facilities, or the community with or without home health care. Index hip fractures were defined as the first inpatient hospital admission during the study period, identified by *International Classification of Diseases, Ninth Revision* (*ICD-9*) or *International Statistical Classification of Diseases and Related Health Problems, Tenth Revision* (*ICD-10*) diagnosis codes corresponding to femoral neck or intertrochanteric fractures of the hip.^14^ We excluded those living in long-stay nursing home residences prior to admission.^15^ The fall-relatedness of each hip fracture was determined using external injury codes (E-codes), consistent with prior work.^16^ The creation of the sample cohort is detailed in the eAppendix in Supplement 1.

### Calculating Days at Home

Our primary outcome for this study was a count of days at home over 12 months after hip fracture discharge. Days at home has been validated against functional status and quality of life, suggesting it measures domains of importance for older adults.^17,18^ For each patient-month during the study period, we calculated home time by subtracting the total days spent in hospitals, skilled nursing facilities, or long-term care settings; under hospital observation; and in the emergency department from the days alive. Hospitalizations, including short stays, long-term acute care, and inpatient rehabilitation stays, were identified through MEDPAR data. Both short-stay and long-stay nursing facility days were verified using MDS data; emergency department observation stays were extracted from Medicare outpatient claims files using revenue codes specific to each setting, in line with prior methods.^19^ Days at home counts were calculated monthly, from 6 months before to 12 months after the hip fracture index admission. If a patient died during any follow-up month, days at home data for subsequent months were designated as missing for the remainder of the follow-up period—death was the only mechanism for missing days at home data.

### Neighborhood Deprivation

Neighborhood disadvantage was defined using the Area Deprivation Index (ADI), a census-based measure incorporating 17 indicators related to poverty, education, housing, and employment derived from the American Community Survey.^20^ Each Medicare beneficiary was linked to the ADI by the 9-digit zip code for their residence at the time of the hip fracture, if their zip code was assigned to a residential address and not a post office box. Consistent with prior research showing threshold effects of neighborhood deprivation at extremes of poverty,^20,21^ we divided the cohort into the 10th percentile or lower (categorized as the least deprived), the 11th to 89th percentile (moderately deprived), and the 90th to 100th percentile (the most deprived). Area Deprivation Index values are unavailable for extremely sparsely populated areas or areas where more than 33% of residents live in group quarters due to missing underlying data; therefore, beneficiaries in these areas were excluded.

### Covariates and Descriptive Variables

Patient age, sex, and race and ethnicity (single item; Black, Hispanic, White, and other [American Indian or Alaska Native, Asian, Native Hawaiian and Pacific Islander, or those who identified as belonging to multiple racial subgroups]) were extracted as documented in the Medicare Beneficiary summary file. Data on race and ethnicity were collected for descriptive purposes. To characterize multimorbidity, we used the Elixhauser Comorbidity Index (range, 0-30, where a higher score indicates more comorbidities) extracted from *ICD-9* and *ICD-10* diagnosis codes present in Medicare inpatient, outpatient, durable medical equipment, and postacute care claims during the 6 months before hip fracture.^22^ The Elixhauser Comorbidity Index was chosen due to common use in hospital systems, known associations with mortality, and weaker but significant associations with functional status among older adults.^23^ A diagnosis of Alzheimer disease and related dementias (ADRD) was identified in Medicare claims using the Chronic Conditions file, which required 1 or more claims for ADRD during a 2-year lookback period.^24^ We also captured county-level rates of facility characteristics, including hospital bed numbers (as a proxy for hospital resources) and teaching hospital status from the Medicare Provider of Services File and county-level rates of nursing home bed availability per 100 000 population as a proxy for nursing home capacity.

### Statistical Analysis

Data analysis was conducted from January 2023 through September 2025. The demographic and clinical characteristics of the individuals with hip fracture were described for the overall sample and stratified by ADI category. To visualize trends, we initially plotted the unadjusted monthly mean count of days at home with 95% CIs in the 12 months after hip fracture, stratified by ADI status, with the count in the month prior to admission included for reference. Monthly counts were used to evaluate time trends in recovery patterns. We subsequently evaluated the adjusted association between ADI status and each monthly count of days spent at home after hip fracture using observation-specific weighted generalized estimating equations (GEEs) with a log-link function and an autoregressive correlation structure.^25,26^ In brief, the probability of missing data due to death at each month was estimated by a pooled logistic regression, incorporating variables such as age, sex, Elixhauser Comorbidity Index score, the number of days at home in the prior month, dementia status, and time; this probability was then used to generate inverse weights applied to the adjusted regression models. Weighted GEEs were adjusted for age, sex, surgery type, Elixhauser Comorbidity Index scores, and total number of days at home in the 6 months before the fracture (as a proxy for prefracture health status), as well as hospital bed size, nursing home bed availability at the county level, and teaching hospital status. The model additionally included time terms (month and quadratic transformation of month after the fracture) to account for the nonlinear trajectories. This weighted GEE modeling approach helps reduce bias due to death by providing an estimate of the mean difference in days at home across ADI groups under the assumption all individuals remained alive through 12 months of follow-up. The adjusted incidence rate ratios (IRRs) estimated in our models represent the proportional differences in post–hip fracture days at home across ADI status. The absolute estimated mean numbers of days at home by ADI status were then estimated holding covariates at their mean values or reference values. Cases with missing county-level covariates (1% [n = 568]) were not used in the fully adjusted analysis. In all cases, statistical significance was defined as an IRR that did not cross 1. Data analysis was conducted with SAS Studio, version 9.04 (SAS Institute Inc).

## Results

The final analytical sample included 561 976 person-months among 52 012 older adults (mean [SD] age, 82.2 [8.1] years; 38 360 women [73.8%] and 13 652 men [26.2%]; 1591 Black participants [3.1%], 594 Hispanic participants [1.2%], 48 243 White participants [93.1%], and 1379 participants [2.7%] of other race or ethnicity) with a fall-related hip fracture admission between July 1, 2010, and December 31, 2019 (Table). In addition, 7357 individuals (14.1%) were dually eligible for Medicaid, and 10 382 (20.0%) lived in a rural area. Individuals with hip fracture had spent a mean (SD) of 175.0 (14.8) days at home in the 180 days before the fracture and had a mean (SD) Elixhauser Comorbidity Index score of 6.8 (3.8).

**Table.** Sample Characteristics of Patients

Characteristic	All (N = 52 012)	Least deprivation (ADI ≤10 [n = 5112])	Middle deprivation (ADI 11-89 [n = 42 594])	Most deprivation (ADI 90-100 [n = 4306])
Age, (mean SD), y	82.2 (8.1)	83.3 (8.0)	82.2 (8.1)	81.2 (8.4)
Sex, No. (%)				
Female	38 360 (73.8)	3753 (73.4)	31 437 (73.8)	3170 (73.6)
Male	13 652 (26.2)	1359 (26.6)	11 157 (26.2)	1136 (26.4)
Race and ethnicity, No. (%)^a^^,^^b^				
Black	1591 (3.1)	73 (1.4)	1119 (2.6)	399 (9.3)
Hispanic	594 (1.2)	47 (0.9)	469 (1.1)	78 (1.8)
White	48 243 (93.1)	4585 (90.3)	39 959 (94.2)	3699 (86.1)
Other	1379 (2.7)	375 (7.4)	885 (2.1)	119 (2.8)
Medicaid dual eligible, No. (%)	7357 (14.1)	590 (11.5)	5635 (13.2)	113 (26.3)
Elixhauser Comorbidity Index scores, mean (SD)	6.8 (3.8)	6.5 (3.7)	6.8 (3.8)	7.3 (3.9)
Days at home in the 6 mo prior to hip fracture, mean (SD)	175.0 (14.8)	175.7 (13.3)	175.0 (14.9)	174.1 (16.1)
Hospital stay, mean (SD), d	5.0 (2.9)	5.0 (2.9)	4.9 (2.7)	5.2 (4.0)
ICU stay, No. (%)	2691 (5.2)	216 (4.2)	2172 (5.1)	303 (7.0)
Surgical type, No. (%)				
Total or hemiarthroplasty	19 957 (38.4)	1913 (37.4)	16 387 (38.5)	1657 (38.5)
Internal fixation	32 055 (61.6)	3199 (62.6)	26 207 (61.5)	2649 (61.5)
Nursing home beds per 100 000 population, No. (%)^c^				
<410	17 869 (34.7)	3108 (61.4)	13 964 (33.1)	797 (18.8)
411-643	17 125 (33.3)	1475 (29.2)	14 448 (34.3)	1202 (28.3)
>644	16 456 (32.0)	477 (9.4)	13 729 (32.6)	2250 (53.0)
Hospital beds, No. (%)^d^				
<230	17 154 (33.0)	1333 (26.1)	14 349 (33.7)	1472 (34.2)
231-424	17 412 (33.5)	1672 (32.7)	14 289 (33.6)	1451 (33.7)
>425	17 440 (33.5)	2107 (41.2)	13 950 (32.8)	1383 (32.1)

Abbreviations: ADI, Area Deprivation Index; ICU, intensive care unit.

^a^ Missing observation counts for race and ethnicity was 205.

^b^ All racial and ethnic identities are recorded as presented in Medicare administrative data. Other races and ethnicities included those who were identified in Medicare claims data as American Indian or Alaska Native, Asian, Native Hawaiian and Pacific Islander, or those who identified as belonging to multiple racial subgroups.

^c^ Missing observation counts for nursing home beds was 562.

^d^ Missing observation counts for hospital beds was 6.

Overall, 5112 members of the cohort (9.8%) lived in areas of very low deprivation (ADI ≤10), and 4306 (8.3%) lived in areas with extremely high deprivation (ADI ≥90) (Table). Individuals with hip fracture in the cohort living in the most deprived neighborhoods compared with the least deprived neighborhoods were younger (mean [SD] age, 81.2 [8.4] vs 83.3 [8.0 years]) and more likely to identify as a member of a racial or ethnic minority group (Black or Hispanic, 477 of 4306 [11.1%] vs 120 of 5112 [2.3%]), be dually eligible for Medicaid (113 of 4306 [26.3%] vs 590 of 5112 [11.5%]), live in rural areas (2068 of 4306 [48.0%] vs 60 of 5112 [1.2%]), and live in areas with more nursing home beds per 100 000 people (>644 beds: 2250 of 4306 [53.0%] vs 477 of 5112 [9.4%]). Days at home in the 6 months prior to surgery were comparable across ADI categories.

Over the 12-month recovery period, individuals in the higher deprivation groups had fewer days at home (Figure)—those in the highest deprivation neighborhoods had a mean (SD) of 246.1 (127.5) days at home, those in the middle deprivation group had a mean (SD) of 257.4 (120.4) days at home, and those in the lowest deprivation group had a mean (SD) of 272.2 (109.3) days at home. The mortality rate increased modestly with increasing deprivation levels; there were 692 of 5112 deaths (13.5%) among those in the lowest deprivation group, 6538 of 42 594 deaths (15.4%) in the middle deprivation group, and 697 of 4306 deaths (16.2%) in the highest deprivation group (Table).

**Figure.**
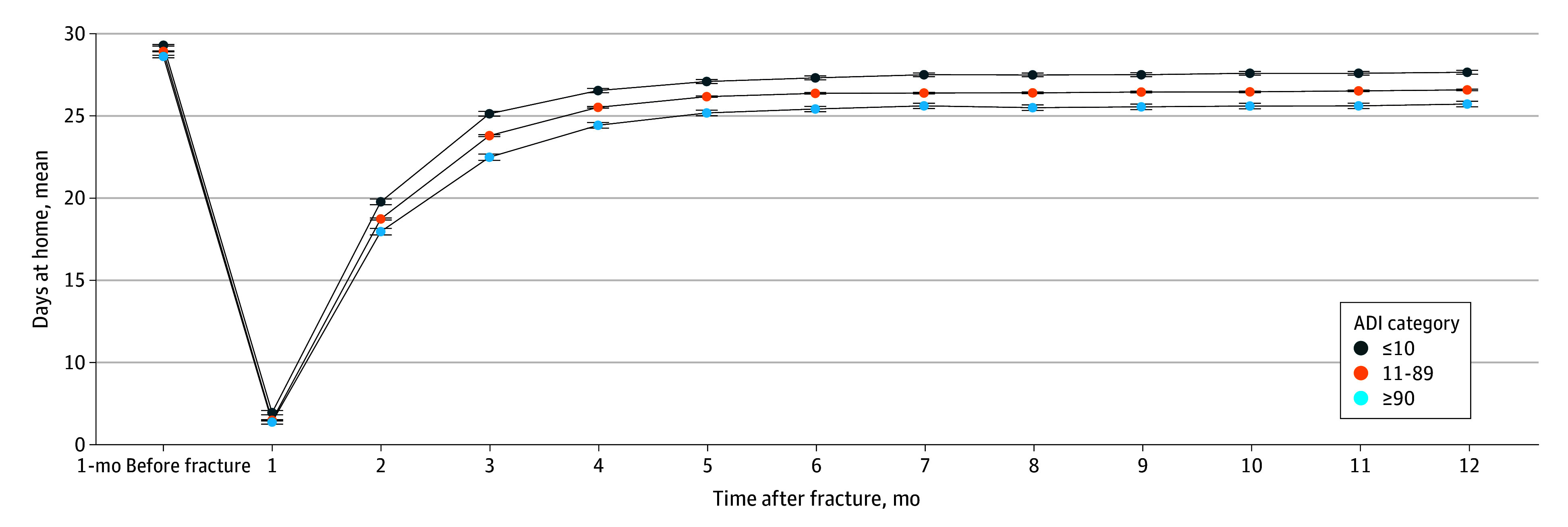
Days Spent at Home in the 12 Months After Fall-Related Hip Fracture Days spent at home in the 12 months after fall-related hip fracture among patients living in the least (Area Deprivation Index [ADI] ≤10), middle (ADI 11-89), and most (ADI ≥90) deprived areas. Days at home in the month prior to hip fracture are shown for reference. Error bars indicate SEs.

In adjusted modeling, differences in days at home by deprivation levels persisted. Relative to the least deprived areas, those in the most deprived areas spent 8.5% fewer days at home (IRR, 0.92; 95% CI, 0.89-0.94). Similar, albeit weaker associations were observed for the middle ADI group compared with the least deprived areas (IRR, 0.95; 95% CI, 0.94-0.97). On an absolute basis, these relative differences translate to mean monthly days at home of 22.6 (95% CI, 22.2-23.0 days at home) for individuals with hip fracture in the least deprived neighborhoods, 21.6 days at home (95% CI, 21.4-21.8 days at home) for those in the middle deprivation group, and 20.7 days at home (95% CI, 20.2-21.1 days at home) for those in the most deprived neighborhoods—over 12 months of follow-up, this is nearly 23 fewer days at home for patients in the most deprived neighborhoods compared with the least deprived neighborhoods. Patients with hip fracture from the least deprived neighborhoods spent, on average, 54.6% of their nonhome days in skilled nursing facilities and 3.4% of their nonhome days in long-term care, patients from moderately deprived neighborhoods spent 53.3% of their nonhome days in skilled nursing facilities and 5.2% of their nonhome days in long-term care, and patients from the most deprived neighborhoods spent 49.2% of their nonhome days in skilled nursing facilities and 6.8% of their nonhome days in long-term care.

## Discussion

In this national cohort study of 52 012 older adults with fall-related hip fracture, we found an inverse monotonic association between neighborhood disadvantage and days at home; those living in the most deprived neighborhoods spent approximately 23 fewer days at home in the year after their injury than those in the least deprived neighborhoods. These findings persisted even after accounting for the competing risk of death among individuals with hip fracture, suggesting that time away from home is not simply a marker of medical vulnerability in this population but instead may reflect resilience in the face of a health stressor.^27^ Overall, these findings suggest that living in a socioeconomically disadvantaged neighborhood is associated with less successful aging in place after a fall-related hip fracture among older adults.

Multiple potential mechanisms could explain our findings. Although our study did not directly examine the pathways associated with neighborhood-level disparities in hip fracture outcomes, existing evidence has consistently shown differences in outcomes by area deprivation across surgical populations^28,29^ and highlights several plausible factors.^30,31^ Environmental barriers in disinvested neighborhoods, such as broken sidewalks, may limit opportunities for safe participation in activities outside the home.^32,33,34^ In addition, residents of these neighborhoods often face reduced access to high-quality postacute rehabilitation care facilities.^35,36^ This lack of access to rehabilitation care may explain why long-term care days increased with increasing levels of neighborhood deprivation in our study. Mental stress associated with living in neighborhoods characterized by higher levels of crime and violence^37^ may compound the stress of a hip fracture, further impairing recovery. Social supports and social interactions, which are associated with postfracture recovery,^38^ may be less available in neighborhoods with greater deprivation.^39^

Neighborhood-level inequities are also rooted in structural factors. Discriminatory policies at local, state, and federal levels have perpetuated residential segregation.^40^ Consequently, residents of disinvested neighborhoods are more likely to identify as members of racial and ethnic minority populations, suggesting that structural racism may be a fundamental factor associated with our findings. In our study, beneficiaries from neighborhoods with greater disadvantage were disproportionately likely to identify as Black, a group previously shown to experience poorer care quality and outcomes after hip fracture.^41,42^ Taken together, these results reinforce the need for multilevel interventional approaches for older adults with hip fractures that address both medical and neighborhood factors.

Given the potential association of neighborhood deprivation with post–hip fracture recovery, changes to practice (including improved collection of social determinants of health data, rehabilitation plans informed by the postdischarge environment, and earlier connection to community resources) may help ameliorate underlying neighborhood level disparities in outcomes. Clinicians are in a prime position to proactively identify the environmental risk factors faced by their patients. But collection of social determinants of health data,^43^ as well as efforts to act on this information after positive screenings, is heterogeneous across hospitals.^44,45,46,47^ Screening for housing needs is completed by an estimated 72% of hospitals in the US, and more than 1 in 4 hospitals do not screen for transportation needs, despite transportation being an important determinant of accessing health care and being less accessible for patients in disadvantaged areas.^48^

Developing comprehensive care pathways to address these neighborhood-specific needs may be a valuable way to improve home time after hip fracture. One potential strategy would be to extend the fracture liaison service model—these liaisons^49,50^ aid in a patient’s navigation of the medical system, enable interdisciplinary communication, and facilitate access to medical and community-based supports. These programs alone have been shown to improve mortality and rates of subsequent fractures.^51^ Although fracture liaison services do not directly intervene on structural disadvantages such as poverty, housing, or employment, they are uniquely positioned to integrate neighborhood context into clinical decision making. By doing so, they can connect patients with trusted community resources and proactively identify and address barriers to high-quality care (eg, transportation challenges that limit access to postfracture clinics).

### Strengths and Limitations

This work has several key strengths that build on prior literature. Our use of the days at home metric—a validated and patient-centered outcome—provides insight into the barriers to aging in place after hip fracture. Furthermore, the novel linkage of 9-digit zip codes with the ADI allowed for a more detailed and granular assessment of neighborhood socioeconomic status surrounding the household in which individuals with hip fracture live than prior analyses using census- or county-level data. This geographic level may be actionable at both clinical and policy levels.

This study also has some limitations. First, while we adjusted our models for a robust array of covariates, important factors such as social supports or transportation barriers are not captured in standard Medicare assessments. Social supports are associated with functional recovery, but it is unclear how some, such as individual-level family supports, may differ in deprived vs less deprived areas. Using this extensive, nationally representative sample of Medicare beneficiaries helps provide generalizable and actionable evidence—future studies could replicate this analysis in datasets with both neighborhood context and caregiver support data to untangle this important question further. Second, we did not include older adults covered by Medicare Advantage, which means that our findings cannot be generalized to this growing group of older adults who are increasingly socially vulnerable and economically disadvantaged.^52^ Third, our use of the ADI may miss some residents in remote rural areas or those in census blocks that are entirely congregate living communities, which, along with our inclusion of only those who were community dwelling, may make our sample skew healthier than the average population of individuals with hip fracture. Fourth, using the ADI to capture deprivation makes it challenging to disentangle the associations of individual-level socioeconomic deprivation and neighborhood-level deprivation; this is because both dual eligibility (Medicare and Medicaid enrollment) and ADI are strongly associated with income and thus lie on overlapping causal pathways. Future work integrating both individual and neighborhood socioeconomic measures could better clarify how each uniquely contributes to inequities in aging in place after traumatic injuries.

## Conclusions

In this cohort study of older Medicare beneficiaries who experienced a fall-related hip fracture, we found that residing in neighborhoods with socioeconomic deprivation was associated with spending fewer days at home in the following year, with those living in neighborhoods with the highest levels of deprivation spending 23 more days away from home compared with those living in neighborhoods with the lowest levels of deprivation. Future studies should seek to develop both immediate and long-term interventions to help patients overcome neighborhood barriers to high-quality aging in place after hip fracture.

## Notes

Supplement 1. **eAppendix.** Creation of the Analytic Cohort

Supplement 2.
Data Sharing Statement
