# Supplementary material for: Neighborhood Deprivation and Days Spent at Home After Fall-Related Hip Fracture
Source: JAMA Netw Open. 2025 Dec 23;8(12):e2549118. doi: 10.1001/jamanetworkopen.2025.49118 (PMC12728656; doi:10.1001/jamanetworkopen.2025.49118)
Supplement: Supplement 1. — eAppendix. Creation of the Analytic Cohort [file jamanetwopen-e2549118-s001.pdf]

Supplementary Online Content

Baginski AM, Braun R, Chen C, et al. Neighborhood deprivation and days spent at home after fall-related hip fracture. *JAMA Netw Open.* 2025;8(12):e2549118. doi:10.1001/jamanetworkopen.2025.49118

eAppendix. Creation of the Analytic Cohort

This supplementary material has been provided by the authors to give readers additional information about their work.

© 2025 Baginski AM et al. *JAMA Network Open.*

eAppendix. Creation of the Analytic Cohort

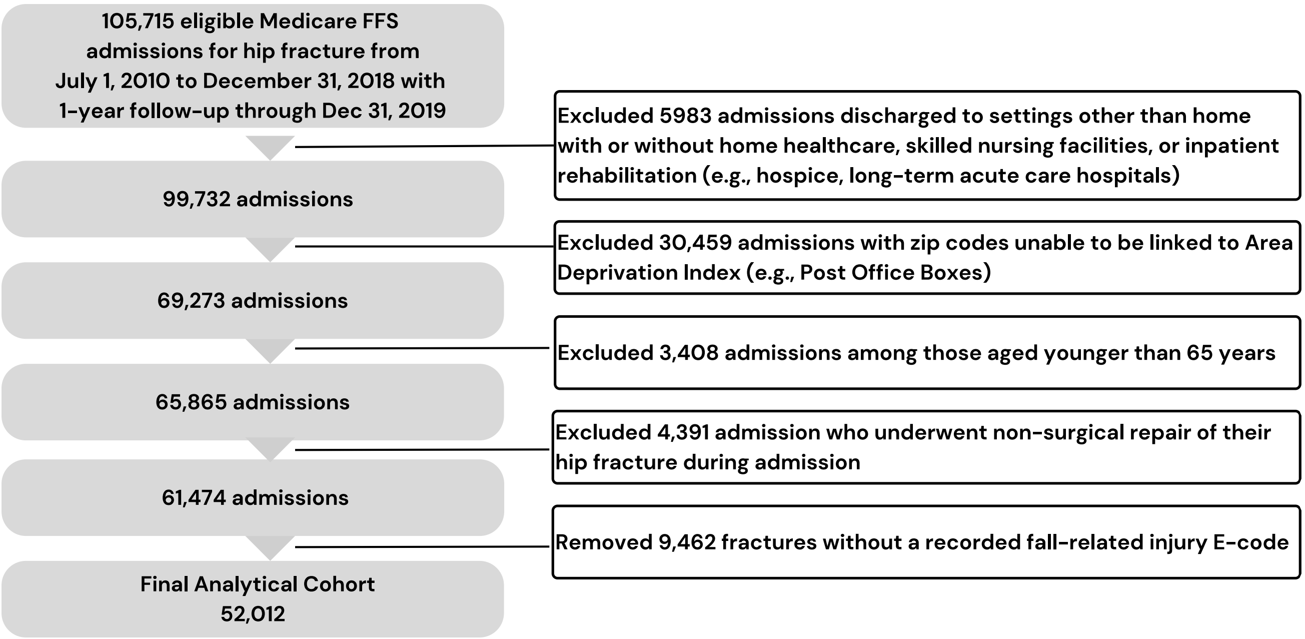

© 2025 Baginski AM et al. *JAMA Network Open.*
