# Supplementary material for: Neighborhood Deprivation and Days Spent at Home After Fall-Related Hip Fracture
Source: JAMA Netw Open. 2025 Dec 23;8(12):e2549118. doi: 10.1001/jamanetworkopen.2025.49118 (PMC12728656; doi:10.1001/jamanetworkopen.2025.49118)
Supplement: Supplement 2. — Data Sharing Statement [file jamanetwopen-e2549118-s002.pdf]

Data Sharing Statement

Baginski. Neighborhood Deprivation and Days Spent at Home After Fall-Related Hip Fracture. *JAMA Netw Open*. Published December 23, 2025. doi:10.1001/jamanetworkopen.2025.49118

Data
Data available: No

Additional Information
Explanation for why data not available: Data used is restricted by data use agreement with CMS
